# U-Net-Based Deep Learning for Simultaneous Segmentation and Agenesis Detection of Primary and Permanent Teeth in Panoramic Radiographs

**DOI:** 10.3390/diagnostics15202577

**Published:** 2025-10-13

**Authors:** Hamit Tunç, Nurullah Akkaya, Berkehan Aykanat, Gürkan Ünsal

**Affiliations:** 1 Department of Paediatric Dentistry, Faculty of Dentistry, Burdur Mehmet Akif Ersoy University, 15100 Burdur, Turkey; berkehanaykanat1994@gmail.com; 2 Department of Computer Engineering, Applied Artificial Intelligence Research Centre, Near East University, Mersin 10, 99138 Nicosia, Turkey; nurullah@nakkaya.com; 3 Schulich School of Medicine and Dentistry, Western University, London, ON N6A 5C1, Canada; gunsal@uwo.ca

**Keywords:** artificial intelligence, agenesis detection, deep learning, mixed dentition, panoramic radiography, maxillofacial radiology

## Abstract

**Background/Objectives**: Panoramic radiographs aid diagnosis in paediatric dentistry, but errors occur. Deep learning-based artificial intelligence offers improved accuracy by reducing overlap-related and interpretive mistakes. This study aimed to develop a U-Net-based deep learning model for simultaneous tooth segmentation and agenesis detection, capable of distinguishing between primary and permanent teeth in panoramic radiographs. **Methods**: Publicly available panoramic radiographs, along with images collected from the archives of Burdur Mehmet Akif Ersoy University Faculty of Dentistry, were used. The dataset totalled 1697 panoramic radiographs after applying exclusion criteria for artifacts and edentulous cases. Manual segmentation was performed by two paediatric dentists and one dentomaxillofacial radiologist. The images were split into training (80%), validation (10%), and test (10%) sets. A U-Net architecture was trained to identify both primary and permanent teeth and to detect tooth agenesis. **Results**: Dental agenesis was detected in 14.6% of 1697 OPGs, predominantly affecting the mandibular second premolars (32.5%) and maxillary lateral incisors (27.6%). Intra- and inter-researcher intraclass correlation coefficients (ICCs) were 0.995 and 0.990, respectively (*p* > 0.05). On the test set, the model achieved a Dice similarity coefficient of 0.8773, precision of 0.9115, recall of 0.8974, and an F1 score of 0.9027. Validation accuracy was 96.71%, indicating reliable performance across diverse datasets. **Conclusions**: The proposed deep learning model automates tooth segmentation and agenesis detection for both primary and permanent dentitions in panoramic radiographs. Its high-performance metrics suggest improved accuracy and efficiency in paediatric dental diagnostics, potentially reducing clinician workload and minimizing diagnostic errors.

## 1. Introduction

Dentists can distinguish between primary and permanent teeth with the clinical examination and not with the use of panoramic radiographs [1]. In paediatric dentistry, panoramic radiographs that has advantages such as patient comfort and simple application are routinely used as complementary tools to clinical examination, providing comprehensive diagnostic information on agenesis and dental development while adhering to the ALARA (As Low As Reasonably Achievable) principle to minimize radiation exposure [2,3]. Errors in radiographic diagnosis can lead to unnecessary increasing the number of appointments, incorrect treatment planning and negative effects on children’s dental development. Although the review of dental panoramic radiographs by another dentist reduces the likelihood of misdiagnosis, the solo practice of dentists makes it difficult for a second dentist to review them [4]. With developing these technologies such artificial intelligence (AI) and machine learning (ML), there’s an important potential to increase diagnostic trueness and success rate of treatment of dental pathologies [4,5,6].

New trends have been provided in healthcare field about form of radiological diagnosis with integrated to AI and ML [7]. AI is explained as algorithms that has the similar pattern with human perceptions to execute definite tasks. AI systems can enhance themselves with repeat on the processed data by them. The behaviours with similar human intelligence can be exhibited by AI such as deciding and visual perception [8]. ML, subfield of AI focuses on developing to algorithms that can learn from tasks to decide or estimation without preprograming and improve to their performance from their program outputs [9]. Deep learning (DL), a sub-set of ML, utilizes artificial neural network to pattern data. The artificial neural networks can process and define data including multiple layers. These abilities of neural networks enable to making tasks that analysis of dental radiographs with using of them. The main difference between DL and ML is DL can modulate the more complex data and learn more effectually from large datasets [10].

Recently, there has been a surge of interest in the application of new computational methodologies that can enhance the analysis of dental images in dental radiography [11]. Image segmentation has been receiving increasing attention over the years [12]. Segmentation is particularly important in clinical settings because it can act as a gatekeeper to the next diagnostic process, that is, performing measurements, analysing changes over time, and linking the tooth to the treatment plan. Segmentation can also help monitor tooth development in paediatric dentistry, where the prediction of eruption timing could prevent impaction [13]. Because timely detection of agenesis has important implications for paediatric populations. Usually, children are referred to a paediatric dentist who initiates longitudinal monitoring throughout the growth period. Agenesis must be followed radiographically over time, as tooth development occurs in stages and some missing teeth may only become apparent later in mixed dentition. Therefore, repeated panoramic radiographs are performed to document the sequential eruption and identify any delayed or absent tooth buds, allowing timely intervention with minimal radiation exposure [14]. At this point, AI-assisted systems stand out as a promising screening tool for the early detection of anomalies such as agenesis [15]. In this context, there is currently an unmet need in automated paediatric dental image analysis by combining the segmentation of both primary and permanent teeth with agenesis detection within a unified framework. The potential clinic for general practitioners then is that they could start with a targeted assessment using a panoramic radiograph. If the dentist establishes a risk for agenesis, however, he or she should refer to a specialist who could use higher-value imaging to confirm the diagnosis. Because traditional methods of manually interpreting radiographs can be time consuming and prone to human error, especially when distinguishing between the stages of tooth development [16]. The advent of AI and DL technologies provides an opportunity to streamline this process, offering consistent and objective analysis that can support dentists in making more informed clinical decisions [17]. By automatic the segmentation and agenesis detection of teeth, these technologies can significantly reduce the workload on dental practitioners, allowing them to focus on more effective patient care [16,17].

Moreover, the implementation of AI and ML in dental radiography aligns with the broader movement towards precision medicine, where treatments are tailored to the individual needs of patients. AI-driven tools can provide detailed insights into the dental development of children, identifying anomalies or patterns that may not be immediately apparent through manual analysis [18]. This not only enhances the accuracy of diagnoses but also enables early intervention, which is particularly important in paediatric dentistry where timely treatment can have long-lasting impacts on a child’s oral health. As the technology continues to evolve, the integration of AI in dental radiography is poised to become a standard practice, paving the way for more efficient, accurate, and personalised dental care [19].

Deep learning techniques, particularly U-Net models, have proven successful in the analysis of medical and dental imaging. U-Net, a convolutional neural network originally designed at the Freiburg University Computer Science Department, is utilized for image segmentation in biomedical image processing research [20,21,22]. U-Net demonstrates a pioneering network for image segmentation, integrating benefits from a fully convolutional network and traditional convolutional networks to support precise recognition of an individual pixel [23]. U-Net is structured as a symmetric feature extraction and decreasing abstraction network with an expansion path that rebuilds the resolution of the feature map, robustly revealing the high-level connections amidst the abstract features [24]. This architecture suits and is the new standard baseline for small or medical and dental image segmentation studies [23,25]. Indeed, the application of U-Net in dentistry is well-documented. Researchers have successfully utilized U-Net-based models for diverse tasks such as the precise segmentation of teeth and jaw structures on panoramic radiographs [26], the detection of carious lesions [27], the assessment of periodontal bone loss [28], and the identification of periapical pathologies [29]. These studies collectively demonstrate the robustness and versatility of the U-Net architecture in automating complex diagnostic tasks in dentistry.

The aim of current study is to develop a deep learning model capable of automatically segmentation and agenesis detection of primary and permanent teeth with a high dice similarity coefficient on panoramic radiographs obtained from one panoramic radiograph units, thereby substantially decreasing the time dentists dedicate to radiological assessments.

## 2. Materials and Methods

### 2.1. Ethical Consideration

In accordance with the ethical principles set forth in the Declaration of Helsinki, Ethics approval was granted by Institutional Ethical Board of Burdur Mehmet Akif Ersoy University. (No. GO 2023/336, approval date 7 June 2023). Informed consent was obtained from all subjects involved in the study.

### 2.2. Research Question (PICO)

The research question (PICO) was “Can a U-Net-based deep learning model reliably perform the automatic segmentation of primary and permanent teeth and detect dental agenesis in paediatric panoramic radiographs compared to manual expert annotation?”

Where the following items are defined:

P (Population): Orthopantomographs (OPGs) of paediatric patients aged 5–15 years, collected under strict inclusion and exclusion criteria.

I (Intervention): Application of a U-Net-based deep learning model for automatic tooth segmentation and agenesis detection.

C (Comparison): Ground-truth manual segmentation and diagnosis performed by two pediatric dentists and one maxillofacial radiologist.

O (Outcome): Accuracy and reliability of segmentation and agenesis detection, evaluated using performance metrics such as Dice Similarity Coefficient (DSC), Intersection over Union (IoU), precision, recall, and F1-score.

S (Study type): Retrospective study based on anonymized panoramic radiographs.

### 2.3. Sample Selection

To ensure the study’s generalizability and eliminate potential biases, the entire database of orthopantomographs (OPGs) from the Faculty of Dentistry was utilized. This included a total of 2065 OPGs, all acquired using two different OPG devices (Planmeca Promax, Planmeca Oy, Helsinki, Finland and Hyperion x5, Myray, Cefla, Imola, Italy), sourced from the archives of the Department of Pedodontics at Mehmet Akif Ersoy University.

### 2.4. Inclusion and Exclusion Criteria

#### 2.4.1. Inclusion Criteria

OPGs of paediatric patients with complete clinical and radiographic records, allowing differentiation between agenesis and other causes of tooth loss.

#### 2.4.2. Exclusion Criteria

Radiographs with dentures, fixed orthodontic appliances, severe motion artefacts, ghost images, edentulous cases, and patients with a history of extractions or trauma leading to tooth loss. Cases of partial tooth loss unrelated to agenesis were also excluded after cross-referencing patient records and dental histories.

After applying these criteria, 368 OPGs were excluded, resulting in a final dataset of 1697 images suitable for the study.

Third molars (wisdom teeth) were excluded from the analysis, as their agenesis is a common developmental variation with limited clinical relevance in paediatric populations.

#### 2.4.3. Description of Measurements

The selected images were initially in DICOM format and were subsequently converted to PNG files. These files were then uploaded to the Computer Vision Annotation Tool (CVAT) for the segmentation process. Manual segmentation of all primary and permanent teeth was performed by two paediatric dentists and one dentomaxillofacial radiologist using the manual drawing semantic segmentation technique. Each object was segmented by delineating its margins through point creation, and these segmentations were used to train the model separately for each structure. The algorithm was developed using the U-Net architecture implemented in Python 3.7. (https://www.python.org/downloads/release/python-370/, accessed on 18 June 2025) The segmentation model was based on a U-Net algorithm composed of seven interconnected encoder–decoder modules. Each encoder block consisted of convolutional layers with batch normalization and ReLU activation, including dilated convolutions (dilation rates = 2, 4, 8) to enlarge the receptive field and capture multi-scale contextual features. The decoder path employed up sampling layers (×2) with corresponding skip connections from encoder stages to preserve spatial details. Feature representations from each stage (R_1_–R_5_) were fused through concatenation before the final output layer to integrate both local and global context. The output layer used a 1 × 1 convolution followed by a sigmoid activation to produce the final segmentation mask, which was up sampled to match the input size. All panoramic radiographs were resized to 512 × 1280 pixels and normalized. Data augmentation techniques such as random rotation (±10°) and horizontal flipping were applied. The model was trained using the Adam optimizer (initial learning rate = 0.0001) and Dice loss function, with a batch size of 16 for 50 epochs. Early stopping and model checkpoint callbacks were employed to prevent overfitting.

**Figure 1:**
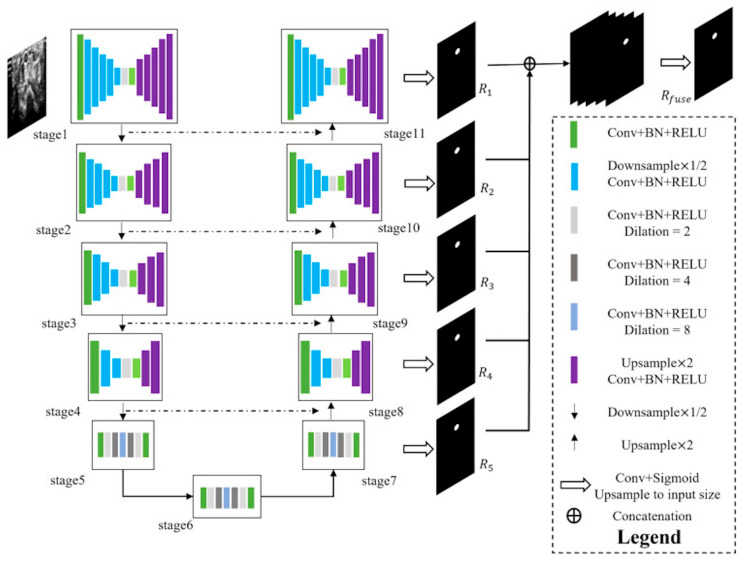
The flowchart of entire process from image input to model segmentation output.

The dataset was divided into training (80%), validation (10%), and test (10%) sets. The model that showed the best performance on the test set was selected for further analysis (Figure 1).

### 2.5. Statistical Analysis

For statistical evaluation, the Dice similarity coefficient (DSC) and accuracy values were computed. The DSC, a measure of similarity between two samples, was used to assess the model’s performance. It is calculated by dividing the total area of the two samples by the size of their overlap. Unlike precision, the DSC accounts for both true positives and false positives, providing a balanced metric that penalizes for both types of errors. The denominator of the DSC includes both the total number of positives, and the positives identified by the model, ensuring comprehensive evaluation of the model’s performance.

## 3. Results

The final dataset consisted of 1697 OPGs from patients aged 5–15 years (mean age: 10.2 ± 2.8 years). The sample included 812 males (47.8%) and 885 females (52.2%). A total of 50,910 annotated regions of interest (ROI) were included in this study. Among these, 24,437 ROI (48.0%) corresponded to primary teeth, and 26,473 ROI (52.0%) corresponded to permanent teeth. This distribution indicates a nearly balanced representation of both tooth types within the dataset, consistent with the mixed dentition stages of the study population. A chi square goodness-of-fit test confirmed that the difference between class proportions was not statistically significant (χ^2^ = 36.4, *p* = 0.07), indicating that the dataset was approximately balanced across classes. Therefore, standard data augmentation techniques were sufficient to maintain model generalizability without additional class-balancing adjustments.

**Figure 2:**
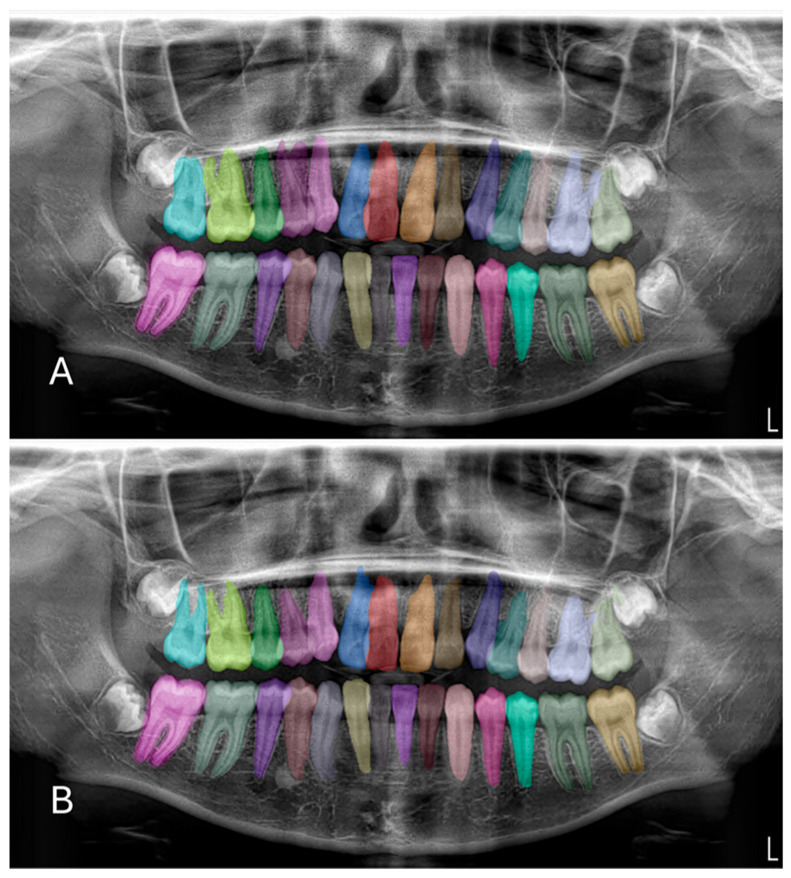
Example images demonstrating the performance for segmentation of permanent teeth on panoramic radiographs between manual annotation by researchers and U-Net based artificial intelligence (AI)-predicted label masks. (**A**) Manual segmentation performed by clinicians. (**B**) Automatic segmentation by the model. In the segmentation figures, each tooth is represented with a distinct colour to visually differentiate regions; however, the model treated all teeth as unified segmentation classes during training.

**Figure 3:**
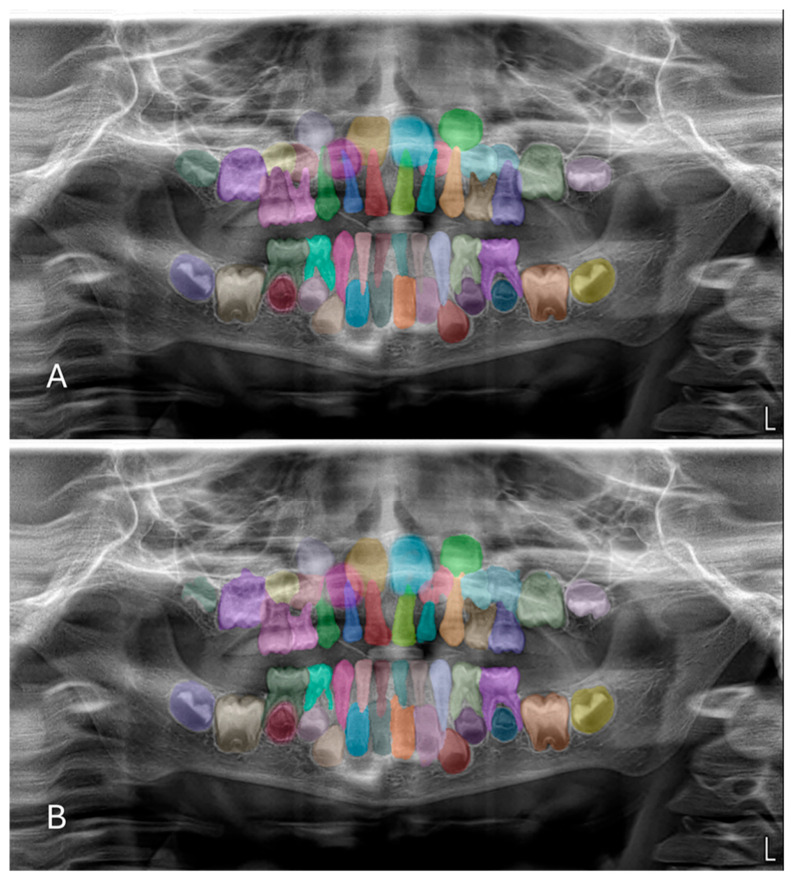
Example images demonstrating the performance for segmentation of mixed dentition on panoramic radiographs between manual annotation by researchers and U-Net based artificial intelligence (AI)-predicted label masks. (**A**) Manual segmentation performed by clinicians. (**B**) Automatic segmentation by the model. In the segmentation figures, each tooth is represented with a distinct colour to visually differentiate regions; however, the model treated all teeth as unified segmentation classes during training.

In the test phase, the U-Net-based deep learning model demonstrated robust segmentation and classification performance of primary and permanent teeth in panoramic radiographs. The Dice similarity coefficient (DSC) was 0.8773, indicating a substantial overlap between the model’s predicted segmentations and the ground-truth annotations (Figure 2 and Figure 3). Concurrently, the intersection over union (IoU) score of 0.8125 reflected the model’s ability to precisely localize target structures. Further supporting its robustness, the model achieved a precision of 0.9115 and a recall of 0.8974, leading to an F1 score of 0.9027. Collectively, these metrics confirm the model’s capacity to accurately delineate teeth while maintaining a balanced trade-off between false positives and false negatives. During the validation phase, the model maintained a high accuracy of 96.71%, indicating consistent performance across a variety of panoramic radiographs. Although the validation DSC (0.8062) and IoU (0.6936) were slightly lower than the test phase metrics—possibly due to dataset variability—the values remained indicative of reliable segmentation. Additionally, the validation loss was recorded at 0.2571, which falls within an acceptable error range for this type of segmentation task. Notably, the peak validation DSC reached 0.8540, while the highest IoU was 0.7556, suggesting that the model shows potential for further refinement.

Out of 1697 OPGs, 248 patients (14.6%) presented with agenesis, corresponding to a total of 387 missing teeth. Most agenesis cases were in the mandibular second premolars (tooth numbers 35 and 45; 32.5%) and the maxillary lateral incisors (tooth numbers 12 and 22; 27.6%). The distribution of agenesis cases is summarized in Table 1.

**Figure 4:**
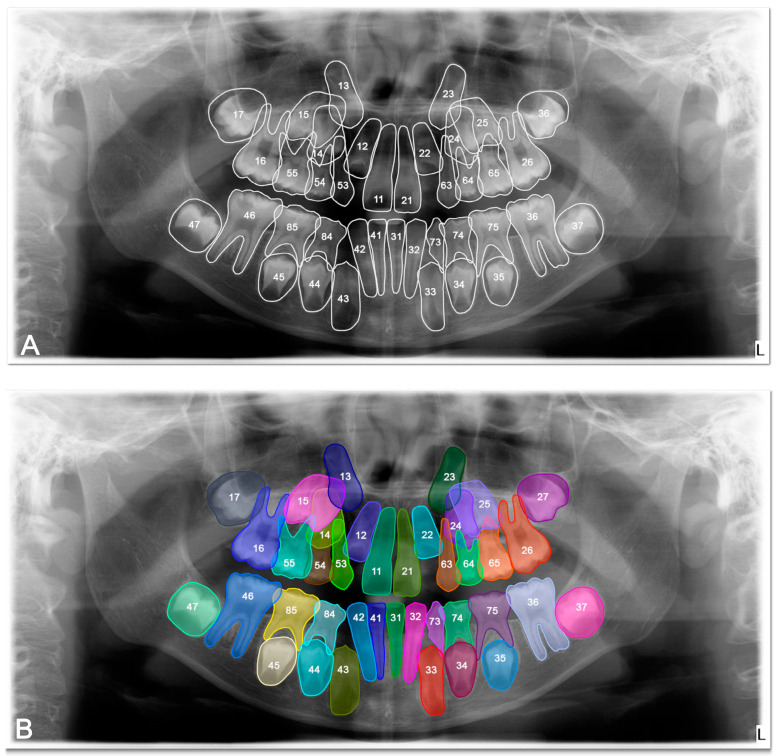
Images demonstrating the performance for tooth agenesis detection on panoramic radiographs between manual annotation by three dentists and U-Net based artificial intelligence (AI)-predicted label masks. (**A**) Manual segmentation performed by clinicians. (**B**) Automatic segmentation by the model. In the segmentation figures, each tooth is represented with a distinct colour to visually differentiate regions; however, the model treated all teeth as unified segmentation classes during training.

The DSC, precision, recall and F-1 score were 0.8692, 0.9078, 0.8925, and 0.9035 respectively, for tooth detection (Table 2). Examples for the detection of agenesis are shown in Figure 4.

Overall, these results underscore the model’s strong generalization capability and its effectiveness in simultaneously segmenting and detecting agenesis of both primary and permanent teeth.

## 4. Discussion

This paper demonstrated that a U-Net–organized deep learning algorithm can reach high accuracy in segmentation of primary and permanent teeth and, simultaneously, detect agenesis from panoramic radiographs. Integration of segmentation and detection of agenesis within a unified workflow facilitates potential clinical translation to child dentistry. Our network simultaneously performed detection and segmentation and optimized the process. Although simultaneous frameworks reduce the times and computational cost, they have more propensity for false positives in comparison to separate segmentation and detection workflows. We propose future research for systematic comparison of the two approaches to study precision and efficiency-based trade-offs.

In terms of model training, the compilation and optimization parameters used in this study are consistent with recent literature on dental and medical image segmentation. The Adam optimizer with an initial learning rate of 0.0001 has been widely applied due to its stability and efficiency in training convolutional neural networks for radiographic images [25,30]. Similarly, the Dice loss function is preferred in segmentation tasks where class imbalance is present, as it directly optimizes the overlap between predicted and ground-truth masks [31]. The choice of 50 epochs and batch size of 16 aligns with previous dental imaging studies employing U-Net architectures on datasets of comparable size [25,30]. Finally, the integration of early stopping and checkpointing is a standard practice to prevent overfitting and to ensure robust model selection. Collectively, these methodological decisions follow the most up-to-date practices and provide a reliable framework for clinical translation. Overall, these metrics collectively affirm that our DL model not only achieves high accuracy and segmentation quality during training but also generalises well to unseen data. Like previous studies, the high dice coefficients and IoU scores across training, validation, and testing phases validate the model’s reliability and effectiveness in differentiating and segmenting primary and permanent teeth in panoramic radiographs [4,32]. This improvement in diagnostic tools promises to enhance clinical workflows, reduce diagnostic errors, and improve treatment planning in paediatric dentistry.

Among the deep learning techniques used for dental image analyses, three networks: U-Net, Mask R-CNN, and YOLO are the most popularly used for tooth, dental structure and pathologies segmentation and object detection tasks [13,25,33]. Mask R-CNN has a convolutional neural network to identify regions of interest and a region proposal network to investigate each object proposal. You Only Look Once (YOLO) algorithm, is one of the popular CNNs for object segmentation and detection. As the name suggests, the YOLO algorithm can detect objects in a single pass [34]. Firstly U-Net that offered by Ronneberger et al. in 2015 has been extensively utilized for medical imaging segmentation due to its ability to incorporate contextual information while requiring less time and smaller datasets for training [35]. U-Net consists of two sections: the contracting path and the expanding path. The contracting part, named the encoder, incorporates a series of repeated sellers [23]. Additionally, it merges the encoder and decoder by aligning and adjusting the input image to fit the size of feature maps between the encoder and decoder on a layer-by-layer basis, enabling the network to not only classify but also localize objects for segmentation [23,35]. Although U-Net as an artificial intelligence algorithm is shown to have better performance in high-resolution images, in the current study on OPGs, it provided segmentation with an acceptable accuracy with a high DSC value [24]. Compared to other U-Net variants, the basic U-Net model employed in the present study contains fewer parameters, resulting in shorter training times, lower hardware requirements, and a reduced risk of overfitting. Particularly in low-resolution panoramic radiographs, as in the current study, complex U-Net derivatives (e.g., Attention U-Net, Residual U-Net) tend to exhibit overfitting [36]. The availability of labelled data in panoramic radiographs is limited. The basic U-Net demonstrates high accuracy (IoU, Dice) even when trained on small datasets. For instance, a basic U-Net model trained on 200 images achieved a Dice score of 91%, whereas the Attention U-Net, trained on the same dataset, reached only 87% [36]. The simplified “encoder–decoder” architecture of the basic U-Net mitigates gradient instability and shows greater resilience to grayscale inconsistencies commonly found in panoramic radiographs [37]. The basic U-Net more consistently captures edge sharpness and anatomical boundaries (e.g., tooth germ, tooth root) [35]. In contrast, advanced U-Net derivatives may lead to micro-texture loss due to their greater depth. Moreover, the basic U-Net can operate effectively on systems without GPUs, demonstrating high applicability for deployment on edge or mobile devices [38]. Thus, U-Net remains the most practical and computationally efficient approach for clinical translation in paediatric panoramic imaging. The results of this study demonstrate the great potential of deep learning models, particularly U-Net algorithms, in accurately segmenting primary and permanent teeth in OPGs. These results are like previous studies on the segmentation of teeth using U-Net systems [31,39]. In addition to this, DSC value obtained from the present study are consistent with the results of Kaya et al.’s [30] study that performed segmentation using a different algorithm. Another previous study about primary teeth segmentation with using R-CNN method on 421 OPGs showed that F1 score, and precision score were 0.9686 and 0.9571. That study showed that similar results with present study even though only primary teeth were included in that study and number OPGs were lower than current study [16].

While there was no study about tooth agenesis detection with using YOLO algorithm, Mask R-CNN and U-Net model were used for tooth agenesis detection and segmentation in only one study [33]. The U-Net significantly higher success on tooth agenesis and segmentation detection in current study. However, according to results of previous study, in cases of severe tooth agenesis, Mask R–CNN encounters difficulties in performing this segmentation due to gaps and missing teeth. The integration of pre-processing and post-processing steps had contributed to reducing the likelihood of false detections, although some numbering errors persist in previous study [33]. This study suggests the importance of comparison of both previous and present study results and found that the agenesis segmentation dataset is originally distributed in the beginning research only, and limited use of Panoramic Radiograph distributed from different datasets, so it is necessary to compare the performance like sensitivity, specificity, DICE on the further studies.

Several important considerations must be acknowledged. In conducted with previous studies about that primary and permanent teeth segmentation, the study was conducted using radiographs obtained from a single OPG unit, which introduces a single-centre bias too [16,30,31]. This situation implies that the model’s performance may not be fully representative of its effectiveness across different radiographic equipment and settings. Furthermore, while this study and previous studies focused on the segmentation and numbering of teeth, future research could expand the capabilities of AI models to include the detection of other dental conditions, such as caries, periodontal disease, and orthodontic assessments [16,30,32]. Integrating these diagnostic features would create a more comprehensive tool that can assist dentists in various aspects of dental care.

While OPGs are widely used due to their ability to provide a comprehensive view of the jaws and teeth with minimal radiation exposure, they do have limitations. The two-dimensional nature of OPGs can result in distortions and overlapping structures, which can complicate the accurate segmentation of teeth. Additionally, panoramic radiographs do not provide the same level of detail as three-dimensional imaging modalities such as cone-beam computed tomography (CBCT) [40]. However, given the higher radiation doses associated with CBCT, it is crucial to optimise and validate AI algorithms for use with OPG and 2D intraoral radiographs to ensure that they remain the preferred imaging modalities in routine dental practice [16]. But, developing models capable of analysing CBCT images, despite their higher ionising radiation, could offer more detailed and precise diagnostic capabilities.

Despite the promising results, it is essential to address the practical implementation of AI in dental clinics [8]. Developing user-friendly interfaces and integrating AI tools with existing dental practice management software will be critical for widespread adoption [17]. Additionally, training dental professionals to effectively utilise these tools will be necessary to maximise their benefits [6].

Several limitations of the present study warrant consideration. First, although two different panoramic radiography devices were utilised, all data originated from a single institution, which may give rise to centre-specific bias. Accordingly, multi-centre studies are essential to establish the external validity and generalisability of the findings. Second, while patients with a history of extraction or trauma were excluded, subtle diagnostic uncertainties regarding the confirmation of agenesis may still persist. Future investigations should therefore incorporate longitudinal data to achieve a more definitive validation. Third, the inherent limitations of panoramic radiographs as two-dimensional imaging modalities may lead to superimposition artefacts. Although CBCT provides superior diagnostic accuracy, its higher radiation dose restricts its routine application in paediatric populations. Hence, optimising artificial intelligence approaches for two-dimensional imaging remains a critical avenue for future research.

Future research should aim to include multi-centre data to enhance the generalizability of the model and should explore the application of DL algorithms for the automatic detection of age estimation with tooth agenesis. Detecting missing teeth early can significantly impact treatment planning and outcomes in paediatric dentistry. Implementing algorithms that can accurately identify and classify dental anomalies would provide substantial clinical benefits. Future studies should show the whole training and the input sizes of images, and the techniques to fit more data, using data augmentation and normalization. The loss function should be evaluated. It should also provide information on the usage of fully convolutional networks in the state of the art.

## 5. Conclusions

The results of this study showed the effectiveness of the deep learning model developed using the U-Net algorithm for automatic segmentation of deciduous and permanent teeth and agenesis detection in panoramic radiographs. Although the results of the study are satisfactory, it is still important to increase the sample size and conduct multicentre studies. By continuing to refine these technologies and exploring their applications across various imaging modalities and dental conditions, AI has the potential to significantly enhance diagnostic accuracy, streamline clinical workflows, and ultimately improve patient outcomes in paediatric dentistry.

## Figures and Tables

**Table 1:** Distribution of agenesis by jaw and tooth type.

Tooth Type	Maxilla (*n*, %)	Mandible (*n*, %)
Lateral incisor (12, 22)	107 (27.6%)	–
Second premolar (15, 25, 35, 45)	42 (10.8%)	84 (21.7%)
First premolar (14, 24, 34, 44)	11 (2.8%)	17 (4.4%)
Canine (13, 23, 33, 43)	9 (2.3%)	12 (3.1%)
Second molar (17, 27, 37, 47)	15 (3.9%)	18 (4.6%)

**Table 2:** Diagnostic performance of the U-Net system on the test data set images.

	Dice Similarity Coefficient (DSC)	Precision	Recall	F1-Score
Agenesis Detection Model	0.8692	0.9078	0.8925	0.9035
Segmentation Model	0.8773	0.9115	0.8974	0.9027

## Data Availability

The original contributions presented in this study are included in the article. Further inquiries can be directed to the corresponding author.

## Abbreviations

The following abbreviations are used in this manuscript:

AI	Artificial intelligence
OPGs	Orthopantomographs
DSC	Dice similarity coefficient
IoU	Intersection over union
CVAT	Computer vision annotation tool
CBCT	Cone-beam computed tomography
ML	Machine learning
DL	Deep learning
